# Clinical and Laboratory Response of Domiciled Dogs with Visceral Leishmaniasis Treated with Miltefosine and Allopurinol

**DOI:** 10.3390/tropicalmed8100472

**Published:** 2023-10-10

**Authors:** Talita Pereira Vaz, Patrícia Flávia Quaresma, Felipe Dutra Rêgo, Camila Binder Souza, Gilberto Fontes, Célia Maria Ferreira Gontijo

**Affiliations:** 1 René Rachou Institute (FIOCRUZ/MG), Av. Augusto de Lima, 1715, Barro Preto, Belo Horizonte 30190-009, MG, Brazil; talitapvaz@gmail.com (T.P.V.); felipe.rego@fiocruz.br (F.D.R.); camilabinders@gmail.com (C.B.S.); 2 Campus Reitor João Davi Ferreira Lima, Federal University of Santa Catarina, Trindade, Florianópolis 88040-900, SC, Brazil; patricia.quaresma@ufsc.br; 3 Campus Centro Oeste, Federal University of São João del Rei (UFSJ), Sebastião Gonçalves Coelho, 400, Divinópolis 35501-296, MG, Brazil; gfontes@ufsj.edu.br

**Keywords:** *Leishmania infantum*, dog, treatment

## Abstract

Canine visceral leishmaniasis (CVL) remains a significant disease worldwide. In Brazil, its treatment is performed using miltefosine, which has demonstrated promising outcomes in dogs. This study represents the first attempt to treat and monitor dogs with CVL in natural conditions over the course of one year. The dogs were divided into two groups: G1 received miltefosine and allopurinol for 28 days, while G2 received miltefosine for 28 days, followed by allopurinol for one year. The follow-up involved clinical, hematological, and biochemical evaluations, as well as the detection of *Leishmania* DNA in skin and bone marrow samples. By the end of the follow-up, dogs in G2 exhibited improved staging compared to their initial conditions, whereas those in G1 showed worsened staging. *Leishmania* DNA in skin and bone marrow decreased between 6 and 12 months after treatment. Our observations indicate that the treatment using miltefosine reduces the detection of the parasite in the skin and bone marrow for up to one year following its administration. The continuous use of allopurinol contributes to control of the disease in dogs. These findings provide valuable insights into the response of dogs treated in natural conditions, offering essential information for veterinarians and public health authorities.

## 1. Introduction

In Brazil, visceral leishmaniasis (VL) is a chronic, life-threatening disease caused by the protozoan *Leishmania infantum* and characterized by various clinical, epidemiological, biological, and social factors that vary according to the region of occurrence. The disease has been reported in 84 countries, with 79 of them experiencing endemic cases [1]. In the Americas, VL is a zoonotic disease affecting humans and a wide variety of mammals [2]. In 2021, the lethality rate of the disease in the Americas reached 9.5%, the highest since 2012, which was 3.5 times higher than the global rate. During the same year, Brazil accounted for 93.5% of the confirmed cases on the continent [3].

The main reservoir host of *L. infantum* in the context of urban transmission is the domestic dog (*Canis familiaris*) [4]. Canine cases of VL often precede the emergence of human cases [5,6]. Due to intense parasitism in the skin, dogs play a pivotal role in maintaining the parasite in endemic areas, thereby facilitating infection of the insect vectors. Given the significance of domestic dogs in perpetuating the *L. infantum* cycle in urban environments, the Brazilian Ministry of Health has adopted the controversial measure of euthanizing seropositive dogs [5]. This strategy faces resistance among some pet tutors who are unwilling to subject their dogs to this procedure.

Since 2016, the treatment of dogs infected by *L. infantum* has become feasible in Brazil, following the registration of the drug miltefosine, formulated and distributed by Virbac Laboratory as Milteforan^®^ and approved by the Ministry of Agriculture, Livestock, and Food Supply as well as the Ministry of Health [7]. According to the manufacturer, this drug inhibits the penetration of infective forms of *Leishmania* in macrophages and disrupts the signal transduction of the *Leishmania* membrane [8]. Nevertheless, the mechanism of the leishmanicidal action of miltefosine remains poorly understood. In Brazil, miltefosine is not employed for treating VL in humans, allowing for its use in treating infected dogs. However, this treatment is not regarded as a control measure for canine visceral leishmaniasis (CVL) because, despite clinical improvements, the animal remains a reservoir of the etiological agent [9].

In light of the authorization to use miltefosine for the treatment of CVL in Brazil, it is imperative to monitor dogs undergoing this treatment. Understanding its impact on laboratory parameters, as well as its effectiveness in reducing clinical signs and improving the clinical staging of dogs residing in endemic areas in Brazil, is of paramount importance. Furthermore, the influence of natural resistance to miltefosine on CVL treatment should not be underestimated, highlighting the critical significance of monitoring therapeutic responses [10].

Studies have demonstrated a positive response in CVL dogs treated with miltefosine. Some studies have also associated the use of this drug with allopurinol, which, due to its leishmaniostatic activity, contributes to disease control [11,12,13,14]. However, most of these studies have evaluated the treatment of CVL dogs in controlled environments, such as screened kennels with regulated temperatures and a balanced diet, along with deworming and vaccination monitoring. Nonetheless, the response of treated dogs under natural conditions is crucial for providing valuable insights to veterinarians and public health authorities.

The objective of this study was to assess the treatment of domiciled dogs naturally infected with *L. infantum* using miltefosine in combination with allopurinol. We obtained results by monitoring clinical staging, hematological, and biochemical parameters, as well as detecting *Leishmania* DNA in biological samples over a period of one year. To the best of our knowledge, this is the first field trial to evaluate the efficacy of miltefosine in CVL with long-term monitoring while the dogs remain in their usual living conditions with their tutors.

## 2. Materials and Methods

### 2.1. Animals

For the study, 30 domiciled dogs that tested reactive in both the immunochromatographic Dual Path Platform (DPP^®^) and in the enzyme-linked immunosorbent assay (ELISA) for CVL diagnosis were included. These dogs ranged in age from 1 to 14 years and comprised 11 males and 19 females. Their weights ranged from 2 to 32 kg, and they represented various breeds, including one Poodle, two Pinschers, one Labrador, one Brazilian Terrier, and 25 mixed breeds. The dog group was assembled following a serological survey conducted in the municipality of Iguatama, Minas Gerais, Brazil. While Iguatama is considered an endemic area for CVL, no recorded cases of VL in humans have been reported until the end of follow-up (Ethics Committee on the Use of Animals of the Federal University of São João Del Rei—CEUA-UFSJ, under protocol 032/2018).

The tutors were informed of the treatment and expressed their agreement by signing the informed consent form. They remained with their dogs throughout the treatment and follow-up period. All dogs were fitted with collars impregnated with Deltamethrin and Propoxur (Leevre^®^, laboratory Ourofino, Sao Paulo, Brazil), and these collars were replaced after six months in accordance with the manufacturer’s recommendations.

The dogs were subjected to treatment with miltefosine and allopurinol. Miltefosine (Milteforan^®^, laboratory Virbac, Carros, France) was orally administered at a daily dosage of 2 mg/kg over a 28-day period. Allopurinol was administered by the tutors themselves every 12 h at a dosage of 10 mg/kg. Two therapeutic schemes were adopted: Group 1 (*n* = 15): miltefosine and allopurinol for 28 days; Group 2 (*n* = 15): miltefosine for 28 days and allopurinol for one year. The dogs underwent monitoring for a period of 12 months.

### 2.2. Clinical Evaluation

The dogs were submitted to clinical evaluation before treatment and monthly during the follow-up, which was always performed by the same veterinarian. Each clinical sign was assessed and assigned a score on a scale from 0 to 4 based on its presence and severity. The scoring system was adapted from Chagas et al. (2021) [15], considering specific clinical characteristics of the animal group under study. Additionally, the dogs were staged according to the Brasileish protocol (2018) [16], which categorizes the disease into five stages based on serological tests, antibody titration, parasitological examinations, clinical manifestations, and laboratory findings.

### 2.3. Hematological and Biochemical Evaluation

Hemograms, measurements of urea and creatinine concentration, total proteins and fractions assessments, and indirect immunofluorescence assays (IIFA) were conducted prior to treatment and subsequently every three months, totaling five rounds of laboratory evaluations. The IIFA tests were carried out by the ZooGene laboratory, utilizing the Biogene Kit (Biogene, Hong Kong, China) and strictly adhering to the manufacturer’s instructions. All other analyses were carried out by the Zoolabi veterinary laboratory.

### 2.4. Leishmania Species Identification

Healthy skin and bone marrow samples from the animals were collected for the purpose of confirming the infection and identifying the *Leishmania* species. These samples were collected before treatment and at 6 months and 12 months after the beginning of treatment. DNA from skin samples was extracted using the Puregene Cell and Tissue Kit (QIAGEN), while DNA extraction from bone marrow samples utilized the PureLink Genomic DNA Mini Kit (laboratory Invitrogen, Waltham, MA, USA). All procedures were performed according to the manufacturers’ instructions.

The extracted DNA underwent PCR analysis targeting the ITS1 region using the primers 5′ CTGGATCATTTTCCGATG 3′ and 5′ TGATACCACTTATCGCACTT 3′ [17]. In all PCR assays, we included the positive controls consisting of the reference strains of the following *Leishmania* species: *Leishmania amazonensis* (IFLA/BR/67/PH8), *L. braziliensis* (MHOM/BR/75/M2903), *L. infantum* (MHOM/BR/74/PP75), and *L. guyanensis* (MHOM/BR/75/M4147), and the negative control consisting of non-template samples.

PCR products of nearly 350 bp were visualized under ultraviolet light after electrophoresis in 2.0% agarose gel stained with ethidium bromide.

For *Leishmania* species identification, the ITS1 PCR products were digested with the Hae III enzyme (10 U/μL) as described by Schonian et al. (2003) [18]. Restriction profiles were analyzed on a 2% agarose gel stained with ethidium bromide (10 mg/mL) and compared with the previously described *Leishmania* reference strains.

### 2.5. Statistical Analysis

The normal distribution of the data was assessed using the Shapiro-Wilk test. To compare proportions, the chi-square test and the Fisher test were employed, with analysis performed utilizing the Openepi 3.01 program. To compare medians, the Kruskal-Wallis and Mann-Whitney tests were performed, with the GraphPad Prism 8.0.1 software (San Diego, CA, USA) being used to compare the medians. The selected statistical significance level was 5% (*p* < 0.05).

## 3. Results

### 3.1. Clinical Evaluation

Among the 30 dogs included at the beginning of the study, 3 (10%) were found to be asymptomatic, while 27 (90%) exhibited CVL signs. The clinical signs most frequently observed before treatment initiation were onychogryphosis and lymphadenomegaly, which were diagnosed in 70% of the dogs. The frequencies of clinical signs documented before treatment, at 6 months, and at 12 months following treatment initiation are detailed in Table 1. It is noteworthy that in group 2, one dog died before completing the Milteforan^®^ (laboratory Virbac, Carros, France) treatment, so this group remained with 14 dogs.

**Figure 1:**
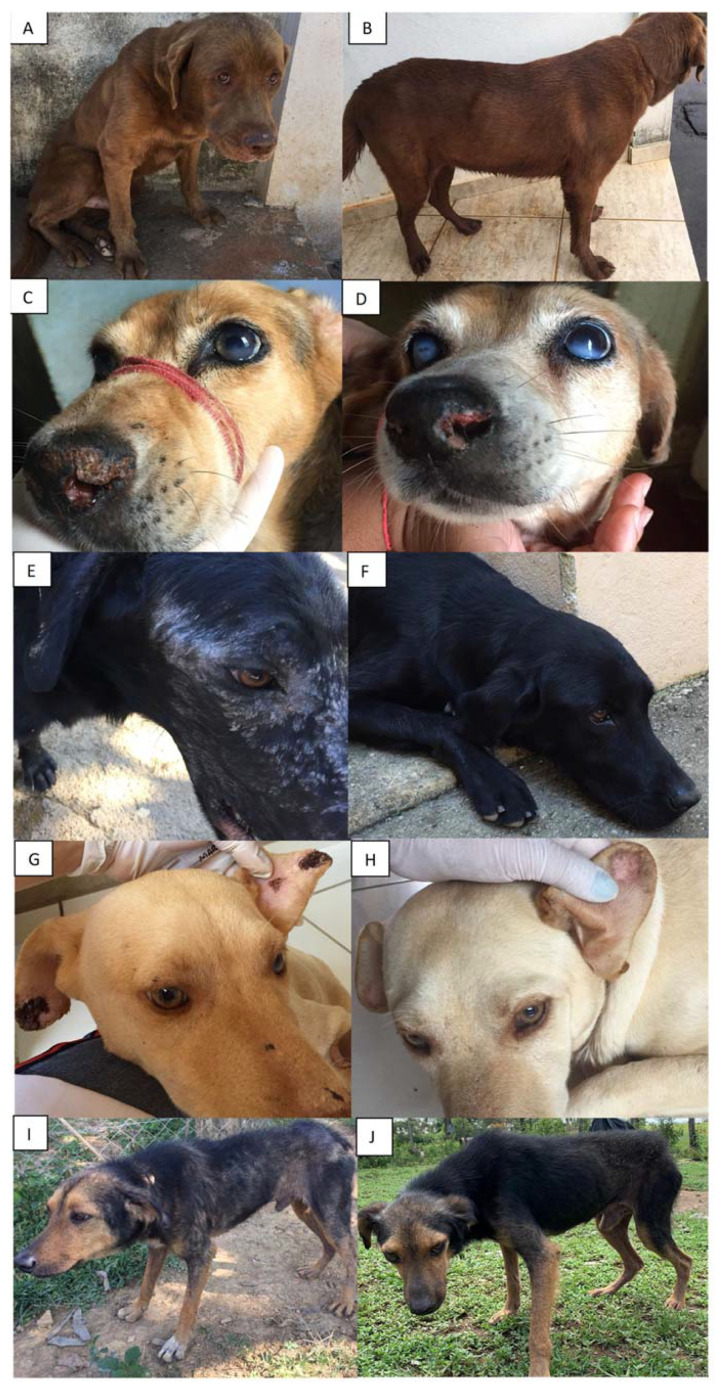
Dogs prior to treatment (**A**,**C**,**E**,**G**,**I**) and up to six months after the beginning of treatment for canine visceral leishmaniasis (**B**,**D**,**F**,**H**,**J**). Figure (**E**,**F**) show a dog of G1, and the remaining figures are of G2 dogs.

Improvement in CVL clinical signs was observed in both groups (Figure 1). The median clinical score of G1 dogs decreased from 6 (IQR: 2–14) before treatment to 1 (IQR: 0–3) at the end of the follow-up period, and this change was not statistically significant (*p* > 0.05). The clinical score of G2 dogs had a median of 12 (IQR: 4.5–25) before treatment and exhibited a significant reduction to 1 (IQR: 0–5.75) eight months after the initiation of treatment (*p* = 0.0074) and to 1.5 (IQR: 0.75) (*p* = 0.0146) at the conclusion of the study.

The clinical staging of dogs yielded different results in the two therapeutic groups. Stage III was the most prevalent in both groups prior to treatment. While there was an improvement in the staging of dogs in both groups six months after the treatment initiation, by the end of the study, all dogs in G1 had regressed to a worse stage when compared to their initial results. In contrast, G2 dogs displayed better staging than before treatment at the end of the follow-up period. The staging of dogs in G1 and G2 before treatment, at 6 months, and at 12 months after treatment initiation is detailed in Table 2.

Lethality in G1 was 33% (5/15) and 29% (4/14) in G2. There was no significant difference between them (*p* = 0.57).

### 3.2. Biochemical and Hematological Evaluation

The most frequent finding before treatment was hypoalbuminemia, which was present in 80% of the dogs. At the end of the study, a significant decrease in the proportion of dogs with this condition was observed (35%) (*p* = 0.003566). This reduction was observed in both therapeutic groups, but it was only statistically significant in G2 (*p* = 0.04886).

Hyperglobulinemia was the second most prevalent finding, affecting 73% of dogs before treatment. A significant decrease in the number of affected dogs (35%) was observed three months after treatment initiation (*p* = 0.01151), probably due to the significant reduction in dogs with this condition in G2 (*p* = 0.04919). At six months after the start of treatment, there was an increase in the number of dogs with hyperglobulinemia compared to the previous evaluation (*p* = 0.002749). This increase was observed in both groups, and in G1, all dogs presented this condition from the sixth month after treatment initiation until the end of follow-up.

Renal function was assessed through urea and creatinine concentrations. At the end of the follow-up period, G2 exhibited an increase in the proportion of dogs with elevated levels of urea and creatinine, although without statistical significance. None of the dogs in G1 had high creatinine concentrations at the end of the study, but 20% of them had elevated urea levels at the end of follow-up.

Anemia was observed in 60% of dogs before treatment. This rate decreased to 39% at six months after treatment initiation but significantly increased again at the end of the study (75%) (*p* = 0.03992). Both groups showed an increase in the number of dogs with anemia from six months onward. By the end of the follow-up period, all G1 dogs presented anemia, while in G2, the rate was 50%.

Before treatment, 50% of the dogs had thrombocytopenia, and six months later, this number significantly decreased to 8.7% (*p* = 0.002644). There was a reduction in this condition in both therapeutic groups, but the reduction from before treatment to six months after treatment was significant (*p* = 0.01495) only in G1. Prior to treatment, leukocytosis was observed in 20% of the dogs (6/30). Only one dog had neutrophilia; the others had lymphocytosis. At the end of the study, no dog showed increased leukocyte counts. Leukopenia was observed in 13% of dogs before treatment. The difference in leukocyte counts during the monitoring of dogs was not statistically significant (*p* > 0.05).

The median value of anti-*Leishmania* antibody titers, as determined by IIFA of the dogs’ sera, decreased six months after treatment in both therapeutic groups, coinciding with the improvement of the dogs’ staging. The decrease was significant only in G1 (*p* = 0.0095). At the end of the follow-up period, antibody titers increased in both groups, and the median antibody titers in G1 were the same as those observed before treatment, while in G2, the value was lower than that found at the beginning (Table 3). The biochemical, hematological, and serological data of individual animals can be found in the Supplementary Materials Tables S1, S2 and Figure S1.

### 3.3. Identification of Leishmania Species

Before treatment, *Leishmania* DNA was detected in the skin of all dogs and in the bone marrow of 90% of them. There was a reduction in the number of positive skin samples at both 6 months and 12 months after the initiation of treatment. By the end of the follow-up period, only 15% of dogs remained positive in skin samples, all of which belonged to G1. No dog in G2 exhibited skin parasitism at the end of the study.

Positivity in bone marrow samples decreased significantly at 6 months and at 12 months after the start of treatment (*p* = 0.005413 and *p* = 0.00002089, respectively). The decrease was observed in both therapeutic groups, but significance was achieved only in G2 at 6 months (*p* = 0.02828) and at 12 months (*p* = 0.00006955) after treatment initiation.

Infection with the species *L. infantum* was confirmed in all 30 dogs included in the study, as detected in skin and/or bone marrow samples.

## 4. Discussion

The treatment of dogs with CVL in Brazil was allowed since 2016, with the approval of Milteforan^®^ (laboratory Virbac, Carros, France) [7]. Before the release of this drug, all CVL-seropositive dogs should be euthanized, regardless of the stage of disease progression. Among the concerns about authorization for the treatment are the absence of a proven parasitological cure for the dog, which may continue to be a reservoir of the etiological agent of the disease, and the risk of the emergence of resistance to miltefosine, considering the few drugs available for the treatment of human leishmaniasis. In view of these concerns, studies on the response of dogs with CVL treated in Brazil are very important.

In the present study, Milteforan^®^ (laboratory Virbac, Carros, France), in association with allopurinol, was used in domiciled dogs naturally infected by *L. infantum*. Improvement in the clinical staging of the dogs was observed six months after treatment in both groups. At the end of the study, only the group of dogs that used allopurinol during the entire follow-up period showed improvement in staging.

Studies have demonstrated a good clinical response in dogs naturally infected by *L. infantum* and treated with miltefosine alone or in combination with other drugs [11,13,14,19,20]. Our results showed that associating Milteforan^®^ with the continuous use of allopurinol contributes to controlling clinical signs of the disease.

The most frequent clinical sign in dogs was onychogryphosis, and this finding is consistent with Teixeira et al. (2020) [21], who state that onychogryphosis is one of the most commonly found clinical signs and is significantly associated with CVL. The median clinical score showed a significant reduction only in G2 (group that continued to use allopurinol) at 8 and 12 months after the beginning of treatment. Ramos et al. (2023) [19] did not use allopurinol in dogs and observed a gradual but not significant reduction in clinical scores of dogs treated with miltefosine at 3 months after treatment.

The results of Nogueira et al. (2019) [13] show a significant reduction in the clinical score of dogs treated only with miltefosine up to 12 weeks after treatment. In our study, the group that did not continue the use of allopurinol showed no significant decrease in clinical scores in the evaluations performed in the follow-up period. Andrade et al. (2011) [11] treated dogs with miltefosine alone and observed total remission of clinical signs in half of the dogs after 24 months of treatment. In our study, only the group that used allopurinol continuously showed a reduction in the median clinical score one year after the beginning of treatment. It is noteworthy that Andrade et al. (2011) [11] did not observe a parasitological cure and even noticed a progressive increase in parasite load, especially six months after the start of treatment. Therefore, the authors do not recommend the treatment of dogs as a CVL control measure.

Hematological evaluation showed that 60% of dogs had anemia before treatment, values similar to those found by Abbehusen et al. (2017) [22] in experimentally infected dogs. Meléndez-Lazo et al. (2018) [23] observed anemia in 62.7% of naturally infected dogs, as in our study. According to Maia and Capino (2018) [24], anemia is the most common hematological abnormality in dogs with clinical leishmaniasis. This alteration may arise from a decrease in erythropoiesis caused by intense bone marrow parasitism or from reduced erythropoietin production due to chronic kidney failure as a result of the disease. Another factor that may contribute to this outcome is hemolysis caused by erythrocyte sequestration in the liver and spleen, enlarged by the inflammatory response to infection [24,25,26]. The dogs that continued the use of allopurinol for one year were less affected by anemia. This may have occurred due to the maintenance of a lower parasite load, avoiding the intense parasitism of the bone marrow, and the decrease in erythropoiesis.

Thrombocytopenia was present in half of the seropositive dogs before treatment, which corroborates studies showing that it is a common finding in CVL [24,27,28]. This alteration may occur due to vasculitis, platelet destruction after renal failure, or the presence of antiplatelet antibodies [27,28]. Six months after treatment, a decrease in thrombocytopenia was observed, which may be related to a depletion in the inflammatory response due to the probable reduction in parasite load.

Although neutrophilia is a common finding [24], our study found only one dog with this alteration before treatment, and at the end of the follow-up, no animal had a high leukocyte count, which may be due to the decrease in inflammatory response caused by treatment.

Dogs with VL are expected to present hyperglobulinemia and hypoalbuminemia as variations in the concentration of these proteins [29]. In our study, these were the most frequent laboratory alterations, agreeing with the findings of Andrade et al. (2011) [11]. At the end of treatment, we observed a significant decrease in dogs affected by hypoalbuminemia, which suggests that these animals did not have proteinuria or significant liver damage, factors that would prevent the increase in this protein even after treatment [30,31].

Dogs that continued to use allopurinol for one year (G2) were significantly less affected by hyperglobulinemia three months after the start of treatment. G1 dogs also showed a reduction in hyperglobulinemia, but without significance. According to Torres et al. (2011) [12], protein changes require three to four months after treatment to normalize. Disease severity has been correlated with hyperglobulinemia [23,31,32]. Thus, the significant reduction of this disorder in dogs that continued the use of allopurinol suggests that the drug contributes to controlling the manifestations of the disease in the animal. At six months after treatment, all dogs that did not continue using allopurinol had this alteration, and it remained until the end of the follow-up period, once again suggesting that allopurinol contributes to controlling the severity of the disease. It is vital, however, to consider potential side effects of allopurinol treatment in dogs, such as the risk of urolithiasis and xanthine calculi formation in the urinary tract, which could lead to chronic renal failure in prolonged allopurinol therapy for dogs [12,33,34]. Therefore, careful monitoring of serum and urine biomarkers during treatment should be duly considered [35].

An increase in urea and creatinine concentrations was observed one year after the initiation of treatment. Azotemia can be observed only when most of the nephrons are dysfunctional, which happens in the advanced stage of the disease [36]. Nephropathy caused by CVL occurs by immune complex deposition in the glomeruli, leading to glomerulonephritis [37]. Increased urea and creatinine levels in the dogs at the end of the study indicate that the disease was not fully controlled.

Positivity for *L. infantum* in the skin samples decreased at 6 months and 12 months after the beginning of treatment in both therapeutic groups, and no dog that continued the use of allopurinol presented the parasite in skin samples at the end of follow-up. Nogueira et al. (2019) [13] suggest that miltefosine therapy reduces the parasite load on the skin of treated dogs, which may have contributed to a drop in positive results in the samples tested in our study. According to Chagas et al. (2021) [15], the skin should be used to monitor *L. infantum* infections in dogs with different clinical stages because it is a tissue with a high parasite load and the access point of the vector. Thus, the treatment protocol, used by associating miltefosine with allopurinol, was effective in reducing the detection of *Leishmania* in the skin of animals, suggesting that it may contribute to reducing the risk of transmission to vectors.

We observed a significant reduction in positivity for *L. infantum* in bone marrow samples at 6 months and 12 months after the start of treatment. Our results are in agreement with Ramos et al. (2023) [19]. They detected a downward trend in the parasitic load in the bone marrow of dogs treated with miltefosine three months after treatment. A different result was found by Andrade et al. (2011) [11], who observed that all bone marrow samples from miltefosine-treated dogs were positive at 3, 6, and 24 months after treatment. Therefore, the treatment seems promising to reduce the detection of *Leishmania* in the bone marrow of treated dogs, but it does not guarantee the complete elimination of the parasite, requiring adjustments to a more effective protocol.

Antibody titers decreased six months after the beginning of treatment and rose again until the end of the follow-up period. In G1, the reduction observed at six months was significant, but at the end of the study, the dogs had the same value as the initial median. G2 dogs had reduced antibody titers at the end of follow-up, but without significant difference. Ramos et al. (2023) [19] also observed a decrease, without significance, in antibody titers three months after treatment with miltefosine. Ayres et al. (2022) [14] did not observe a reduction in antibody titers, but the evaluation was carried out 28 days after treatment with miltefosine and allopurinol. According to Paltrinieri et al. (2016) [31], a decrease in antibody titers is expected six months after treatment, but in dogs living in endemic areas, the complete clearance of antibodies is unlikely. In relapses and the active phase of infection, high antibody titers can be observed; thus, antibody titration can be a useful tool in monitoring dogs under treatment, but not in the short term [11,38].

Treatment and follow-up of the dogs in our study were performed at the tutors’ homes. Natural factors of a pet’s routine, such as nutrition, environment, vaccination, deworming, and treatment of other diseases, were not controlled. As this was a heterogeneous dog sample that has not undergone any standardization, our study reflects the reality of the treatment routinely performed by veterinarians in CVL-endemic areas in Brazil. Most of the available studies were conducted under controlled conditions, excluding the impact that routine variables may have on the animal’s response to treatment. Thus, our study allows us to understand the real effect of the treatment on the dog’s natural conditions, bringing very important information to the veterinary clinician and to the public health authorities. However, considering that dogs’ living conditions vary in natural environments, additional studies with more animals are needed to confirm this finding.

## 5. Conclusions

In the present study, it was observed that there was an improvement in the staging of the disease after treatment with Milteforan^®^ (laboratory Virbac, Carros, France) and that the continuous use of allopurinol significantly contributed to controlling the manifestations of the infection in dogs. The data indicates that Milteforan^®^ (laboratory Virbac, Carros, France) decreased parasite detection in skin and bone marrow samples up to one year after its use. Despite the clinical improvement observed, we cannot state that there was elimination of parasitism in animals. These results contribute to the knowledge of the response of dogs with CVL treated in uncontrolled environments, residing with their tutors in a CVL-endemic area.

## Figures and Tables

**Table 1:** Percentage of dogs with clinical signs evaluated before, at 6 months and at 12 months after the beginning of treatment.

Clinical Signs	Number of Dogs Affected (%)		
	Prior to Treatment	6 Months after Treatment Beginning	12 Months after Treatment Beginning
Mucous membranes pallor	9 (30)	3 (13)	4 (20)
Keratoconjunctivitis	2 (6.7)	4 (17)	4 (20)
Corneal opacity	5 (17)	2 (9)	3 (15)
Blepharitis	7 (23)	0	3 (15)
Uveitis	3 (10)	0	2 (10)
Alopecia	13 (43)	4 (17)	6 (30)
Dermatitis	12 (40)	5 (22)	7 (35)
Hyperpigmentation	5 (17)	1 (4)	2 (10)
Depigmentation	6 (20)	0	2 (10)
Hyperkeratosis	15 (50)	3 (13)	4 (20)
Skin peeling	15 (50)	2 (9)	4 (20)
Paw swelling	0	0	0
Paraparesis	0	0	0
Onychogryphosis	21 (70)	7 (30)	6 (30)
Lymphadenopathy	21 (70)	15 (65)	18 (90)
Lack of appetite	10 (33)	0	1 (5)
Epistaxis	1 (3)	0	0
Vomiting	2 (7)	0	2 (10)
Diarrhea	6 (20)	0	1 (5)
Number of dogs evaluated	30	23	20

**Table 2:** Staging of G1 and G2 dogs according to the Brasileish protocol (2018) [15] before, at 6 months, and at 12 months after the beginning of treatment for canine visceral leishmaniasis.

G1	Prior to Treatment		6 Months after Treatment Beginning		12 Months after Treatment Beginning	
	N° of Dogs	%	N° of Dogs	%	N° of Dogs	%
Stage I	0	0	2	16.7	0	0
Stage II	5	33.3	5	41.7	0	0
Stage III	10	66.7	5	41.7	0	0
Stage IV	0	0	0	0	10	100
Stage V	0	0	0	0	0	0
TOTAL	15	100	12	100	10	100
**G2**	**Prior to Treatment**		**6 Months after Treatment** **Beginning**		**12 Months after Treatment** **Beginning**	
	**N° of Dogs**	**%**	**N° of Dogs**	**%**	**N° of Dogs**	**%**
Stage I	1	7.1	5	45.5	4	40
Stage II	4	28.6	4	36.3	2	20
Stage III	9	64.3	2	18.2	2	20
Stage IV	0	0	0	0	2	20
Stage V	0	0	0	0	0	0
TOTAL	14	100	11	100	10	100

**Table 3:** Median values and interquartile range (IQR) of indirect immunofluorescence assays (IIFA) titers in dogs treated for CVL according to therapeutic groups and follow-up time.

Therapeutic Groups	Evaluation Time		
	Prior to Treatment *	6 Months after Treatment Beginning *	12 Months after Treatment Beginning *
G1	1/320 (1/160–1/320)	1/160 (1/100–1/280)	1/320 (1/280–1/320)
G2	1/240 (1/80–1/320)	1/80 (1/40–1/320)	1/160 (1/80–1/320)

* Median (IQR).

## Data Availability

Not applicable.

## Supplementary Materials

The following supporting information can be downloaded at: https://www.mdpi.com/article/10.3390/tropicalmed8100472/s1, Table S1: Results of hematological and biochemical evaluation of dogs from G1 and G2, before, three, six, nine and twelve months after treatment; Table S2: IIFAT results of G1 and G2 dogs before, three, six, nine and twelve months after treatment. Figure S1: (A) Results of the PCR reaction to verify the presence of *Leishmania* spp. in skin samples collected before treatment. (B) Results of the RFLP reaction to verify *Leishmania infantum* parasitism in skin and bone marrow samples collected before treatment.
