# Supplementary material for: Clinical and Laboratory Response of Domiciled Dogs with Visceral Leishmaniasis Treated with Miltefosine and Allopurinol
Source: Trop Med Infect Dis. 2023 Oct 10;8(10):472. doi: 10.3390/tropicalmed8100472 (PMC10610677; doi:10.3390/tropicalmed8100472)
Supplement: Supplementary file 1 [file tropicalmed-08-00472-s001.zip › tropicalmed-2610127-supplementary Figure S1.pdf]

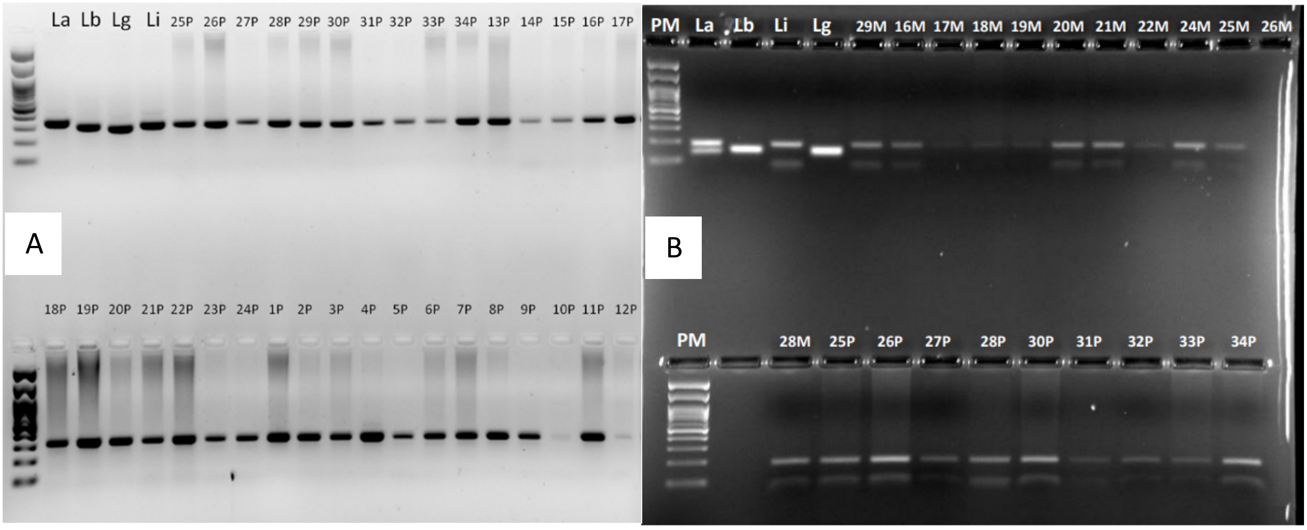

Figure S1: (A) Results of the PCR-ITS1 to verify the presence of *Leishmania spp*. in skin samples collected before treatment. (B) Results of the RFLP-ITS1 to verify *Leishmania infantum* parasitism in skin and bone marrow samples collected before treatment.
